# Immersive clinical learning using simulation and its impact on academic performance, satisfaction, self-confidence, and anxiety among pediatric nursing students

**DOI:** 10.3389/fmed.2026.1742360

**Published:** 2026-05-07

**Authors:** Majeda M. El-Banna, Eman Alshawish, Waqas Sami, Moattar Raza Rizvi, Intima Alrimawi

**Affiliations:** 1 College of Nursing, QU-Health Sector, Qatar University, Doha, Qatar; 2 Faculty of Nursing, An-Najah National University, Nablus, Palestine; 3 Department of Pre-Clinical Affairs, College of Nursing, QU Health, Qatar University, Doha, Qatar; 4 Faculty of Allied Health Sciences, Santosh Deemed to be University, Ghaziabad, India; 5 Georgetown University, Berkley School of Nursing, Washington, DC, United States

**Keywords:** anxiety, clinical competence, nursing education, self confidence, simulation training, student satisfaction

## Abstract

**Background:**

Clinical education forms the foundation of nursing competence, yet access to equitable and high-quality pediatric clinical placements remains limited in many regions. Immersive clinical learning through simulation offers students a safe, authentic space to apply knowledge, build confidence, and manage anxiety before caring for real patients. This study examined the association between simulation-integrated clinical learning and academic performance, satisfaction, self-confidence, and anxiety among undergraduate pediatric nursing students, comparing it with traditional hospital-based training.

**Methods:**

A cross-sectional study was conducted among 191 third-year undergraduate nursing and midwifery students enrolled in a pediatric nursing course. Students participated in either simulation-integrated clinical training (*n* = 148) or traditional hospital training only (*n* = 43). Data were collected using a structured survey that included academic indicators (GPA, theory, and clinical grades), the National League for Nursing’s Satisfaction and Self-Confidence in Learning Scale, and the Generalized Anxiety Disorder-7 (GAD-7) tool. Analyses employed the Mann–Whitney *U* test, *t*-test, and correlation analyses. Logistic regression was used to examine predictors of group allocation, with effect sizes and 95% confidence intervals reported (*p* < 0.05).

**Results:**

Between-group comparisons showed no statistically significant differences in GPA (*p* = 0.066; *r* = 0.18), pediatric theory (*p* = 0.053; *r* = 0.19), or anxiety scores (*p* = 0.322, *g* = −0.17). Satisfaction and self-confidence were strongly correlated (*r* = 0.674, *p* < 0.001). Effect sizes for most academic outcomes were small, with confidence intervals indicating imprecision around group differences. Higher theory exam scores predicted lower anxiety (*β* = −0.186, *p* = 0.008). Students with higher GPAs were more likely to be allocated to simulation-integrated training (OR = 2.918, *p* = 0.047), indicating a potential for selection bias.

**Conclusion:**

Immersive simulation-integrated clinical learning was associated with academic and psychological outcomes comparable to traditional hospital-based training within this institutional context. However, the non-randomized design, and evidence of GPA-related group allocation suggest the presence of selection bias, and findings should therefore be interpreted cautiously. Further randomized or longitudinal studies are required to establish causal relationships and evaluate the long-term impact of simulation-based clinical education.

## 1 Introduction

Clinical education stands at the core of undergraduate nursing preparation, allowing students to translate theoretical learning into real-world practice. As healthcare systems grow more complex and the demand for pediatric services rises (1), traditional hospital placements often struggle to accommodate sufficient student learning opportunities, especially in resource-constrained environments. These clinical experiences are essential for cultivating critical reasoning, psychomotor skills, and professional identity, ensuring that graduates are capable of delivering safe and competent care (2). Yet, nursing programs worldwide continue to grapple with serious challenges that hinder consistent, equitable, and high-quality clinical exposure. Limited numbers of trained clinical educators, variability among placement sites, and inconsistent teaching practices all contribute to disparities in student learning (3, 4). Such inconsistencies raise concerns not only about clinical preparedness but also about fairness and educational equity in the evaluation of student competence (5, 6). In regions affected by political or resource constraints, such as Palestine, these obstacles are even more significant, demanding innovative and flexible alternatives to traditional clinical teaching (7).

The difficulties become even more pronounced in pediatric nursing, where students face unique barriers to experiential learning. Hospitals often restrict student participation in pediatric wards due to infection control protocols, privacy concerns, and the sensitive nature of caring for children (8). A shortage of pediatric-specialized instructors further compounds these limitations (9). These conditions can limit opportunities for hands-on practice, leading to gaps in communication, assessment, and family-centered care skills core competencies essential for pediatric nursing (10). Such constraints can erode confidence and leave students feeling unprepared for independent clinical decision-making, making it imperative to explore innovative teaching approaches that ensure equitable learning opportunities.

Simulation-based education (SBE) has emerged as one such pedagogical solution that offers structured, repeatable, and safe clinical experiences. Through the use of high-fidelity manikins, virtual platforms, standardized patients, and realistic scenarios, simulation allows students to practice technical and critical-thinking skills without compromising patient safety (11–13). This instructional model integrates debriefing and guided reflection, aligning closely with adult learning and experiential education theories. Particularly in pediatric nursing, simulation may provide a controlled and emotionally safe environment to rehearse complex interactions, refine technical skills, and build confidence before entering real clinical settings.

For pediatric nursing students, immersive simulation experiences can bridge many of the gaps left by traditional clinical placements. They help learners practice communication with families, manage emergencies, and make ethical and developmental considerations unique to children (14, 15). The controlled repetition of these scenarios reinforces psychomotor accuracy and conceptual understanding, improving both confidence and competence (15). Meta-analytic evidence further supports that high-fidelity simulation enhances nursing students’ knowledge, judgment, and long-term retention of skills (16, 17). These benefits suggest that pediatric simulation may function as a useful educational supplement in preparing practice-ready graduates.

In recent years, the emotional and psychological dimensions of simulation have attracted increasing scholarly interest. Learning outcomes are now viewed as multidimensional encompassing cognitive mastery as well as emotional readiness. Student anxiety, confidence, and satisfaction have been shown to strongly influence academic success and future clinical performance (18). Simulation can provide a psychologically safe space that encourages experimentation and reflection without fear of patient harm (19). Yet, some studies reveal that the realism of simulation and performance evaluation can, at times, elevate stress or anxiety levels, especially when debriefing or faculty support are insufficient (20). These mixed findings highlight the importance of understanding how simulation affects both learning and emotional well-being within specific clinical contexts.

The effectiveness of simulation depends not only on its duration but also on the design quality and emotional climate of the experience. While some researchers emphasize the value of extended simulation hours, others stress that scenario realism, instructor engagement, and alignment with course outcomes are more influential in determining learning impact (19). Pediatric nursing simulations must also address the unique sensitivities of working with children and families areas often underrepresented in general simulation curricula (21). This becomes especially significant in low-resource or conflict-affected regions such as Palestine, where simulation serves as both an educational innovation and a pragmatic substitute for unavailable clinical sites.

Previous studies conducted in Palestine have shown that nursing students generally value supportive clinical supervision and technologically enhanced learning environments (22–25). Research has highlighted the need for expanded simulation infrastructure and faculty training to enhance engagement and skill acquisition (26–28). However, most of this evidence relates to adult or emergency nursing contexts. A few notable studies have examined satisfaction and confidence in high-fidelity adult nursing simulations (26, 29, 30), and although pediatric simulation has been explored in prior research, including studies demonstrating improvements in knowledge, clinical skills, and self-efficacy (14, 15), applications examining academic performance alongside emotional outcomes such as confidence and anxiety remain limited within the undergraduate Palestinian context.

Given this evidence gap, the present study sought to evaluate how simulation-integrated clinical learning influences academic performance, satisfaction, self-confidence, and anxiety among undergraduate pediatric nursing students in Palestine. We hypothesized that simulation-integrated training would yield comparable academic outcomes while enhancing self-confidence and satisfaction without increasing anxiety. The findings are intended to provide localized, evidence-based insights for the design of immersive and emotionally supportive pediatric simulation curricula.

## 2 Materials and methods

### 2.1 Study design

This study adopted a cross-sectional design to compare the influence of simulation-integrated clinical learning with traditional hospital placements on students’ academic performance, satisfaction, self-confidence, and anxiety. The primary outcome of interest was academic performance (GPA and pediatric course grades). Anxiety levels (GAD-7) were designated as secondary outcomes, while satisfaction and self-confidence measures were considered exploratory outcomes specific to the simulation-integrated group. The cross-sectional approach was chosen for its ability to describe existing relationships between variables within a defined period. It offers efficiency in terms of time and resources and allows for the identification of potential associations that can later be examined through longitudinal or experimental designs (31).

### 2.2 Participants

The study population comprised third-year undergraduate students enrolled in the Bachelor of Nursing and Midwifery pre-licensure programs at a university-affiliated health sciences institution. All participants were registered in the pediatric nursing course during the semester in which data were collected. Students were eligible if they were in their third year, taking the pediatric course for the first time, had completed the full clinical training schedule (simulation-based or traditional), and provided informed consent. Students repeating the pediatric course, withdrawing consent, or submitting incomplete questionnaires were excluded to ensure a homogenous first-time cohort and avoid bias. Out of 260 initially eligible students, 18 repeaters were removed, leaving 242 who consented to participate. Of these, 51 students were excluded due to incomplete questionnaires, resulting in complete data for 191 students and a final response rate of 79%. Of the final sample, 148 students (77.5%) completed simulation-integrated clinical training, which combined simulation and hospital-based practice, while 43 students (22.5%) participated in traditional hospital-based training only. Baseline demographic and academic characteristics were compared between groups to assess potential selection bias. Differences were observed for age and hospital days (*p* < 0.05), while other characteristics showed no statistically significant differences (*p* > 0.05).

### 2.3 Sampling

A complete enumeration sampling method was employed to include all eligible students and reduce sampling-related bias. Group allocation (simulation-integrated clinical training vs. traditional hospital-based training) was non-random and determined by clinical placement capacity and administrative scheduling. Students assigned to the simulation-integrated group were able to replace unavailable hospital clinical hours with structured simulation sessions, whereas students in the traditional group completed hospital-based training only. Because site availability varied across rotation periods, randomization was not feasible; however, both groups completed equivalent total clinical hours as mandated by the curriculum. Although allocation was not based on predefined academic criteria or student preference, subsequent analysis revealed that students with higher GPA were significantly more likely to be assigned to the simulation-integrated group. This suggests the presence of unintended or implicit selection mechanisms and indicates a potential for selection bias that should be considered when interpreting the findings.

### 2.4 Setting and duration

The pediatric clinical training spanned 21 days, amounting to a total of 168 h. Clinical training included both hospital-based placements and simulation-integrated activities. Simulation activities comprised both laboratory-based sessions conducted in the university simulation center and *in situ* simulation sessions implemented within the hospital setting. The majority of simulation hours were delivered in the simulation laboratory, with a smaller proportion conducted in situ. Hospital-based clinical sessions were conducted daily from 7:00 a.m. to 2:00 p.m. with a 30-min break, whereas simulation sessions were held from 8:00 a.m. to 1:00 p.m. with a 15-min break. Simulation hours replaced unavailable hospital placements but maintained parity in total learning time, thereby offering a balanced exposure to essential pediatric competencies through both real and simulated experiences. For the purposes of analysis, ‘one simulation day’ was defined as a structured five-hour instructional session (8:00 a.m.–1:00 p.m.) comprising pre-briefing, scenario implementation, and facilitated debriefing. Simulation exposure was therefore operationalized both as total hours and as the proportion of simulation hours relative to the overall 168-h clinical training period. Debriefing was conducted immediately following each scenario using a structured reflective format led by the clinical instructor, focusing on clinical reasoning, decision-making processes, teamwork, and emotional processing. The debriefing phase typically lasted 20–30 min and followed a guided discussion framework to reinforce learning objectives and promote psychological safety. Simulation exposure was quantified both as the number of simulation days and as total simulation hours, and the proportion of simulation hours relative to the total 168-h clinical rotation was calculated for each participant.

Simulation scenarios addressed core pediatric competencies, including management of respiratory distress, pediatric medication administration and dosage calculation, fluid and electrolyte monitoring, febrile child assessment, and family-centered communication. High-fidelity manikins and standard pediatric clinical equipment were used to approximate realistic care environments. Each simulation session involved small groups of approximately 6–8 students supervised by one trained clinical instructor (instructor-to-student ratio approximately 1:6–1:8). A structured debriefing approach was implemented following each scenario, incorporating guided reflection, feedback, and facilitated discussion to reinforce clinical reasoning and emotional processing in alignment with predefined learning objectives.

### 2.5 Instruments

A structured survey-based approach was used to gather data across four key domains: demographic background, exposure to simulation-integrated clinical training, academic performance, and student psychological and perceptual outcomes. Demographic and background information were gathered using a self-reported questionnaire that included items on gender, marital status, household income, place of residence, and English language proficiency. English language proficiency was included because course instruction, theoretical examinations, and simulation scenarios were delivered in English, and variations in proficiency may influence comprehension, participation, and academic performance. Information regarding prior professional healthcare experience was not formally assessed, as participants were pre-licensure undergraduate students and such experience is typically minimal within this cohort. These variables were collected to allow subgroup comparisons and to control for potential confounding effects in the statistical analysis. Previous exposure to simulation-based learning was also not measured as a separate variable; however, all students were enrolled in the same institutional curriculum, which included structured simulation exposure, thereby ensuring a broadly comparable baseline. Students were also asked a single-item preference question: “If given a choice, which clinical training modality would you prefer for pediatric practice?” Response options were (1) hospital-based clinical training, (2) simulation-based clinical training. This study-specific item was administered as a single-response categorical variable (two-category nominal). Simulation exposure was operationalized as the total number of instructional hours completed in simulation-integrated clinical training sessions. In addition to reporting the numbers of simulation days, total simulation hours and the proportion of simulation hours relative to total clinical training were calculated for each participant.

Academic performance was assessed using four key indicators to capture different dimensions of student performance. The first indicator was the cumulative Grade Point Average (GPA), which was self-reported by students and reflects institutional grading procedures applied uniformly across cohorts. The second indicator was the final theory examination score in the pediatric nursing Didactic course, which was expressed as a percentage and reflected the student’s acquisition of theoretical knowledge. The third indicator was the total grade of the Didactic pediatric course. The fourth indicator was the clinical course total grade, which evaluated practical competence within the pediatric course based on performance in real or simulated clinical environments.

This clinical performance was assessed using standardized evaluation forms routinely used by faculty across both hospital-based and simulation-integrated settings to ensure consistency in grading. These forms included structured rubrics covering core competencies such as clinical decision-making, technical skill execution, communication, and professional behavior. Evaluations were conducted by trained supervisors using consistent scoring criteria across both training modalities; these supervisors were not informed of the study hypothesis, enhancing procedural objectivity. The same evaluation rubric and scoring criteria were applied across both hospital-based and simulation-integrated training contexts to maintain grading consistency. Faculty evaluators were experienced clinical instructors routinely assigned to pediatric placements. While complete blinding to training modality was not feasible due to the nature of clinical assignments, instructors were not informed of the study hypothesis. Faculty calibration meetings were conducted at the beginning of the academic year to standardize grading expectations and ensure consistency across evaluators. Although contextual differences between hospital and simulation environments may introduce minor variability in assessment conditions, identical grading criteria were applied across groups to enhance comparability.

Students’ psychological well-being and perceptions of the learning experience were assessed using two validated instruments. The first was the 13-item Satisfaction and Self-Confidence in Learning with Simulation tool developed by the National League for Nursing, which comprises two subscales measuring satisfaction and self-confidence. Items are scored on a five-point Likert scale ranging from “strongly disagree” to “strongly agree.” The tool demonstrates excellent internal consistency with Cronbach’s alpha values of 0.94 for the satisfaction subscale and 0.87 for the self-confidence subscale (32). The second instrument was the Generalized Anxiety Disorder 7-item (GAD-7) scale, which assesses the frequency of anxiety symptoms over the preceding 2 weeks (33). The total score ranges from 0 to 21 and was used both as a continuous measure and to classify anxiety into four severity levels: minimal (0–4), mild (5–9), moderate (10–14), and severe anxiety (15–21). A detailed summary of all study instruments and variables is provided in Appendix A.

### 2.6 Data collection procedure

Data collection was conducted electronically. Survey administration occurred during the final week of the semester, after completion of both theoretical and clinical assessments for the pediatric course, ensuring that anxiety responses reflected overall training experience rather than anticipatory examination stress. Students were invited to participate via institutional email, which included a secure link to the survey along with the consent form and study information. A reminder email was sent 2 weeks after the initial invitation to enhance response rates. The data collection period remained open for 1 weeks to accommodate students’ schedules and final assessments. Cumulative GPA was self-reported (verified against institutional records where applicable) by students within the survey. Pediatric course grades (didactic, final examination, and clinical grades) were retrieved from official course records after final grading was completed. Access to academic records was approved by the Institutional Review Board and limited to variables required for the study. Simulation exposure hours were derived from institutional clinical scheduling records.

### 2.7 Ethical considerations

Ethical approval for the study was obtained from the Institutional Review Board (IRB) of An-Najah National University (Ref No. N.G.S May 2021/16). The research was conducted in accordance with the Declaration of Helsinki. Participation was voluntary, and all students were informed about the study’s purpose, confidentiality safeguards, and their right to withdraw at any stage. Electronic consent was obtained before participation, and all responses were anonymized.

### 2.8 Data analysis

Data were analyzed using IBM SPSS Statistics version 29.0. Descriptive statistics, including means, standard deviations, frequencies, and percentages, summarized participant characteristics and scale scores. The Mann–Whitney *U* test and two-sample *t*-test were used to assess group differences for non-normally and normally distributed variables, respectively. Correlations were evaluated using Pearson or Spearman coefficients, depending on data distribution, with bootstrap-adjusted 95% confidence intervals applied to improve reliability. Group comparisons for categorical variables such as anxiety categories were analyzed using the Fisher–Freeman–Halton exact test. To quantify baseline imbalance between groups, standardized mean differences (SMD) were calculated in addition to *p*-values. Given the unequal distribution of participants between groups, SMDs were used to assess the magnitude of baseline imbalance. Adjusted analyses were performed to account for potential bias arising from non-random group allocation. Multivariable linear regression models were then fitted to adjust for baseline imbalances (age, hospital attendance days, and GPA) when examining primary academic and anxiety outcomes. Adjusted analyses were primarily conducted for the prespecified primary academic outcomes and secondary anxiety outcome. For each primary academic and anxiety outcome, the training group (simulation-integrated vs. traditional hospital-based) was entered as the main predictor, adjusting for age, hospital attendance days, and GPA. To predict anxiety levels, a generalized linear model (GLM) was constructed with educational modality, demographic variables, and academic performance as predictors. Additionally, binary logistic regression with backward elimination examined predictors of simulation participation, including GPA, gender, and English proficiency. Statistical significance was set at *p* < 0.05 for all analyses.

## 3 Results

### 3.1 Participants characteristics

The study included 191 students, with slightly more than three-quarters (*n* = 148, 77.5%) in the clinical and simulation group, compared to the clinical and non-simulation group (*n* = 43, 22.5%). The majority of the students were enrolled in a nursing bachelor program (*n* = 167, 87.4%), and a small number were enrolled in a midwifery program (*n* = 24, 12.6%). Females (*n* = 127, 66.5%) were significantly higher in proportion than males (*n* = 64, 33.5%). Most of the students were single (*n* = 175, 91.6%) as compared to married (*n* = 16, 8.4%). Around 50% of the students earned between 3,000 and 5,000 Israeli New Sheqel (ILS), which is approximately $890 to $1,482. Almost two-thirds of the students resided in the West Bank (*n* = 119, 62.3%), and the remaining students (*n* = 72, 37.7%) were living in other Palestinian areas. English proficiency was predominantly intermediate (*n* = 149, 78.0%), novice (*n* = 20, 10.5%), and proficient (*n* = 22, 11.5%). When asked to indicate their preferred primary clinical training modality, 92.6% of students in the simulation-integrated group indicated a preference for hospital-based clinical training as their primary modality, while 7.4% (*n* = 11) preferred simulation-based training (Table 1).

**Table 1:** Descriptive characteristics of the study students (*N* = 191).

Variables	*n* (%) of total sample (*N* = 191)
Groups	
Simulation-integrated clinical training	148 (77.5%)
Traditional hospital-based training	43 (22.5%)
Bachelor’s program	
Midwifery	24 (12.6%)
Nursing	167 (87.4%)
Gender	
Male	64 (33.5%)
Female	127 (66.5%)
Marital status	
Single	175 (91.6%)
Married	16 (8.4%)
Family income (ILS)^a^	
<3,000	43 (22.5%)
3,000–5,000	93 (48.7%)
>5,000	55 (28.8%)
Residency	
West Bank	119 (62.3%)
Other Palestinian areas	72 (37.7%)
English proficiency	
Novice	20 (10.5%)
Intermediate	149 (78.0%)
Experienced	22 (11.5%)
Preference of practicum modality among simulation-integrated group (*n* = 148)	
Hospital	137 (92.6%)
Simulation	11 (7.4%)

Values are presented as *n* (%) unless otherwise specified.

a Israeli New Sheqel.

GPA appeared approximately normally distributed, with a mean GPA of 2.81 (SD = 0.38). Clinical pediatric grades (median = 88.0; range 63–94) surpassed Didactic grades (median = 75.0; range 45–95). Students attended a median of 6.00 simulation days (range = 2–21) and 14.0 hospital days (range = 4–24). The GAD-7 median score was 7.00 (range 0–21). Based on severity categories, 31.9% of students reported minimal anxiety (0–4), 32.5% mild anxiety (5–9), 29.8% moderate anxiety (10–14), and 5.8% severe anxiety (15–21), approximately 64% of participants falling within the minimal and mild anxiety categories. All anxiety categories, including severe anxiety, were retained in both categorical analyses and continuous GAD-7 score analyses. Satisfaction (median = 3.40; range 1.0–5.0) and self-confidence (median = 3.50; range 1.0–5.0) scores reflected positive perceptions of the simulation-integrated learning experience.

### 3.2 Students’ satisfaction and self-confidence in simulation-based learning

Results presented in Table 2 show that students expressed moderate satisfaction with simulation-based teaching methods, with 44–49% agreeing or strongly agreeing on the effectiveness of instructional approaches, materials, and alignment with learning preferences. However, considerable proportions of respondents remained undecided (27–37%) or expressed disagreement (20–22%), highlighting variability in perceptions of pedagogical efficacy. This level of agreement is lower than typically reported, where simulation-based learning is associated with high satisfaction. This may be due to integration with hospital training, preference for traditional clinical experiences, and variability in simulation exposure (2–21 days). In contrast, self-confidence outcomes demonstrated stronger consensus in domains tied to instructor support and learner agency (55–62%) endorsed the utility of instructor-provided resources, personal responsibility for learning, and the ability to seek assistance. Confidence in content mastery and simulation’s role in skill development was comparatively lower, with only 44–47% agreeing or strongly agreeing on these items.

**Table 2:** Student perceptions of satisfaction and self-confidence in simulation-based learning (simulation-integrated group, *n* = 148).

Survey items	SD^a^	D^a^	UN^a^	A^a^	SA^a^	Total (*N*)
Satisfaction with current teaching						
Teaching methods helpful and effective	11 (7.4%)	21 (14.2%)	51 (34.5%)	51 (34.5%)	14 (9.5%)	148
Variety learning materials and activities promote learning	6 (4.1%)	23 (15.5%)	55 (37.2%)	42 (28.4%)	22 (14.9%)	148
Enjoyed how instructor taught simulation	17 (11.5%)	15 (10.1%)	43 (29.1%)	49 (33.1%)	24 (16.2%)	148
Teaching materials motivating, helped learning	11 (7.4%)	23 (15.5%)	43 (29.1%)	56 (37.8%)	15 (10.1%)	148
Way taught suitable to way learn	8 (5.4%)	27 (18.2%)	41 (27.7%)	54 (36.5%)	18 (12.2%)	148
Self-confidence in learning						
Confident mastering content	6 (4.1%)	24 (16.2%)	53 (35.8%)	49 (33.1%)	16 (10.8%)	148
Confident simulation covered critical content	7 (4.7%)	22 (14.9%)	54 (36.5%)	54 (36.5%)	11 (7.4%)	148
Confident developing skills, knowledge to perform clinical tasks	10 (6.8%)	16 (10.8%)	52 (35.1%)	55 (37.2%)	15 (10.1%)	148
Instructor used helpful resources	5 (3.4%)	11 (7.4%)	40 (27.0%)	71 (48.0%)	21 (14.2%)	148
My responsibility to learn what I need to know from simulation	6 (4.1%)	15 (10.1%)	46 (31.1%)	52 (35.1%)	29 (19.6%)	148
I know how to get help when I do not understand concepts	6 (4.1%)	12 (8.1%)	45 (30.4%)	55 (37.2%)	30 (20.3%)	148
I know how to use simulation to learn critical aspects of skills	6 (4.1%)	15 (10.1%)	47 (31.8%)	64 (43.2%)	16 (10.8%)	148
Instructor’s responsibility to tell me what I need to learn of simulation content	5 (3.4%)	12 (8.1%)	31 (20.9%)	63 (42.6%)	37 (25.0%)	148

a SD, strongly disagree; D, disagree; UN, undecided; A, agree; SA, strongly agree. Responses were measured using a 5-point Likert scale, percentages are calculated within row totals.

### 3.3 Correlation between satisfaction, confidence, and simulation exposure

**Figure 1:**
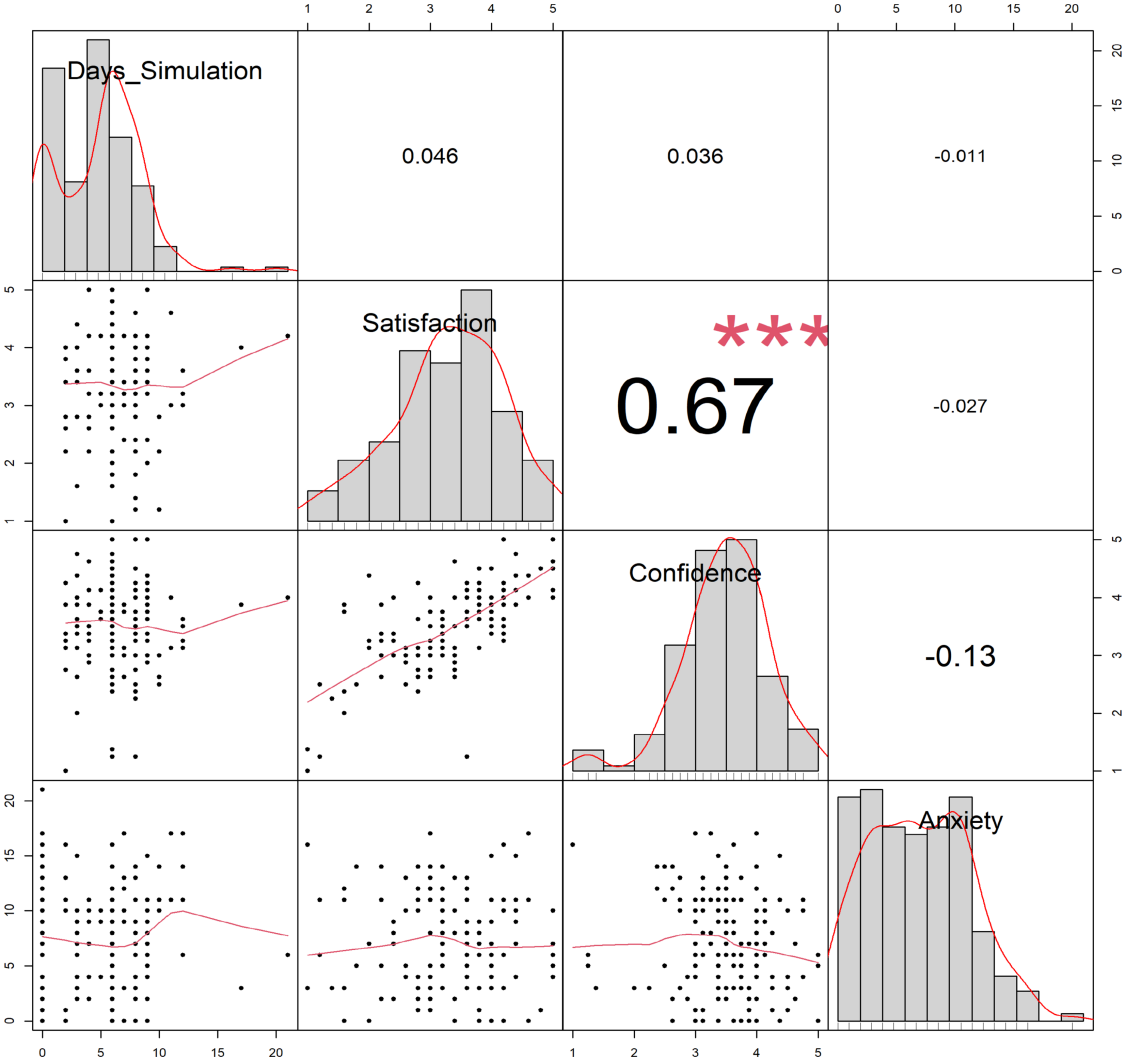
Pairwise correlation matrix of simulation days, satisfaction with learning, self-confidence in learning, and anxiety scores. Diagonal cells display variable distributions using histograms with kernel density overlays. Lower triangle panels show scatterplots with loess smoothed lines, while the upper triangle panel reports correlation coefficients.

Correlational analyses conducted within the simulation-integrated group (*n* = 148) using bootstrap-adjusted confidence intervals revealed a strong positive relationship between satisfaction with learning and self-confidence in learning subscales [*r* = 0.674, *p* < 0.001, 95% CI (0.574, 0.753)], suggesting that learners who perceived simulation as satisfying also reported greater confidence in their skills. These analyses were limited to the simulation-integrated group, as the Satisfaction and Self-Confidence in Learning Scale is specific to simulation-based experiences and not applicable to the traditional training group. The satisfaction and self-confidence measures were collected exclusively from students exposed to simulation, as the instrument is designed specifically to evaluate simulation-based learning experiences. However, when simulation exposure was modeled as a continuous variable (total simulation hours and number of simulation days), no significant associations were observed with satisfaction (*r* = 0.046, *p* = 0.576), self-confidence (*r* = 0.036, *p* = 0.662), or anxiety scores (*r* = −0.011, *p* = 0.924). Results are presented in Figure 1.

### 3.4 Association between anxiety and sociodemographic variables

The primary analyses focused on between-group differences in academic performance (primary outcomes), with anxiety outcomes examined as secondary measures. When GAD-7 anxiety levels were associated with groups, the results indicated a difference in the distribution of anxiety levels between the two groups, with the simulation group showing a higher proportion of students with mild anxiety (33.8%) and a lower proportion with minimal anxiety (31.8%) compared to the non-simulation group. However, this association was not statistically significant (Fisher–Freeman–Halton Exact test *p* = 0.632).

**Figure 2:**
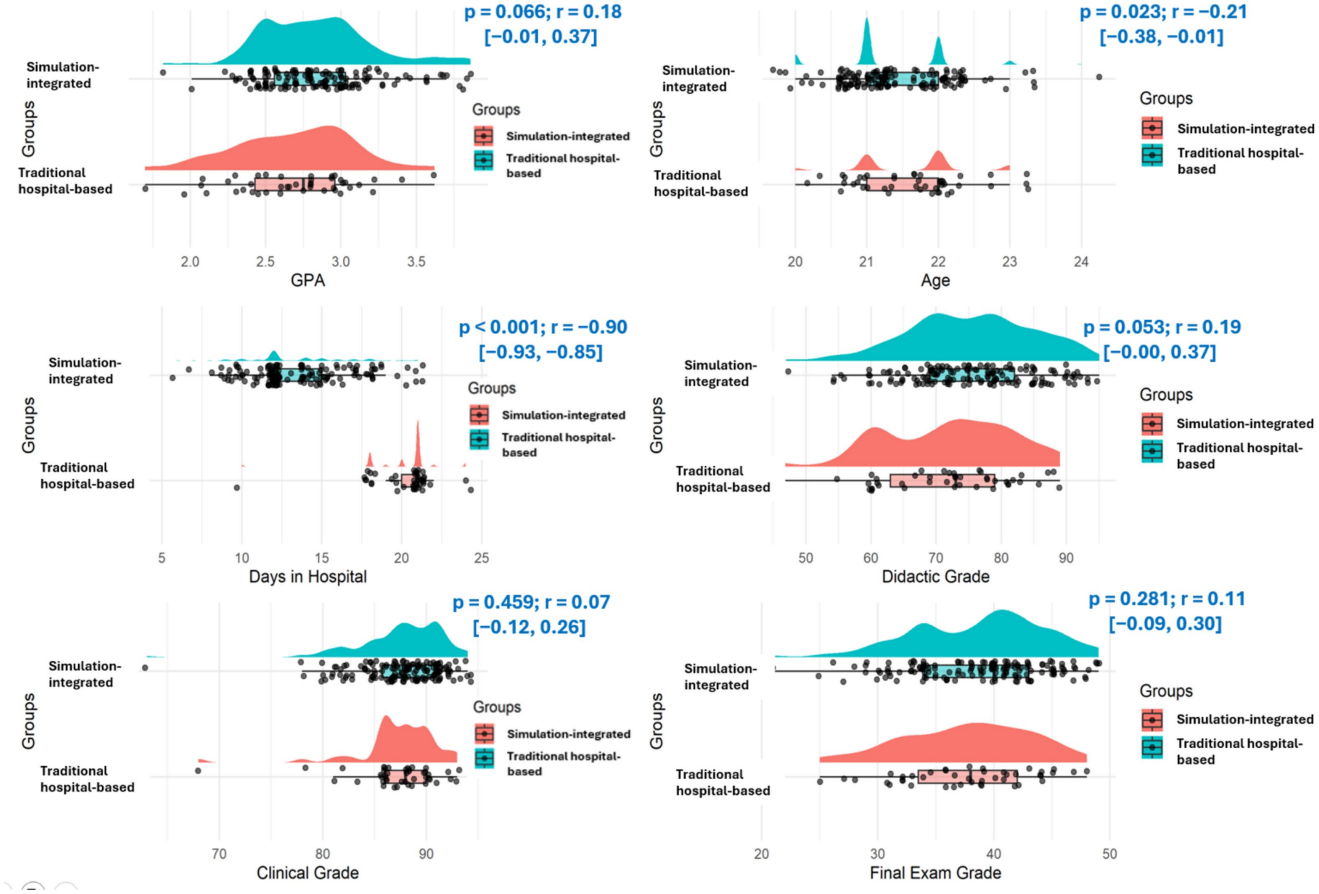
Violin plots with jittered data points comparing academic and demographic characteristics between students who received simulation-integrated training (blue) and those who did not (red). Each panel displays the distribution and spreads for GPA, age, days in hospital, didactic grade, clinical grade, and final exam grade by group. Between-group differences were evaluated using the Mann–Whitney *U* test. Reported *p*-values correspond to the Mann–Whitney test, and effect sizes are presented as rank-biserial correlation (*r*) with 95% confidence intervals shown in brackets. Positive *r* values indicate higher scores in the simulation-integrated group than in the traditional hospital-based group.

The Mann–Whitney *U* test revealed statistically significant differences between the simulation-integrated group and traditional hospital-based group in age [*p* = 0.023, *r* = −0.206, 95% CI (−0.384, −0.012)] and hospital attendance days [*p* < 0.001, *r* = −0.898, 95% CI (−0.930, −0.852)], with the non-simulation group being older (median = 22.0 years vs. 21.0 years) and attending more hospital days (median = 21.0 vs. 12.0). The difference in hospital attendance days was expected and reflects the study design, whereby simulation hours replaced a portion of hospital-based clinical time while maintaining equivalent total clinical training hours across groups. However, no significant differences were observed in GPA [*p* = 0.066, *r* = 0.184, 95% CI (−0.010, 0.365)], total clinical days [*p* = 0.083, *r* = −0.164, 95% CI (−0.347, 0.031)], Didactic pediatric grades [*p* = 0.053, *r* = 0.194, 95% CI (−0.0003, 0.374)], clinical pediatric grades [*p* = 0.458, *r* = 0.074, 95% CI (−0.122, 0.264)], or final exam grades [*p* = 0.280, *r* = 0.108, 95% CI (−0.088, 0.296)]; these results are presented in Figure 2.

Moreover, the GAD-7 score was normally distributed, and an independent samples t-test showed no significant differences in anxiety scores between the two groups [7.91 ± 4.87 vs. 7.13 ± 4.42, *p* = 0.35, mean difference = −0.78, 95% CI (−2.43, 0.87), Hedges’ *g* = −0.17, 95% CI (−0.53, 0.19)]. Across outcomes, effect sizes were small, and confidence intervals generally crossed zero, indicating uncertainty regarding the magnitude and direction of between-group differences.

Standardized mean differences (SMD) were calculated to quantify baseline imbalance between groups. Moderate imbalance was observed for age (SMD = −0.37) and GPA (SMD = 0.40), and substantial imbalance was observed for hospital attendance days (SMD = −2.31). SMD values greater than 0.2 were considered indicative of meaningful imbalance. These imbalances indicate potential selection bias and were therefore accounted for in adjusted analyses. To address potential confounding variables, multivariable linear regression models were conducted adjusting for age, hospital attendance days, and GPA. After adjustment, the group variable was not statistically significant for GPA (*β* = 0.08, *p* = 0.359), Didactic pediatric grade (*β* = 2.84, *p* = 0.081), clinical pediatric grade (*β* = 0.35, *p* = 0.716), final examination grade (*β* = 0.50, *p* = 0.646), or anxiety (*β* = −0.64, *p* = 0.586), indicating attenuation of between-group differences after accounting for baseline characteristics. Taken together, the magnitude of observed effects was small, and the associated confidence intervals suggest that both small positive and negative differences remain plausible. For primary and secondary outcomes, between-group differences are presented with corresponding effect sizes and 95% confidence intervals.

### 3.5 Predictors of anxiety and group allocation

The associations between demographic variables and anxiety severity (GAD-7 categories) were assessed using the Fisher–Freeman–Halton exact test. No statistically significant associations were observed between gender (*p* = 0.395), marital status (*p* = 0.844), family income (*p* = 0.186), residency (*p* = 0.940), English proficiency (*p* = 0.953), or preference for simulation vs. hospital-based clinical training (*p* = 0.837). Results are presented in Table 3.

**Table 3:** Association between GAD-7 anxiety categories and sociodemographic variables (*N* = 191).

Variables	GAD-7 anxiety				*p*-value
	Minimal	Mild	Moderate	Severe	
Gender					
Male (*n* = 64)	16 (32.6%)	24 (31.8%)	19 (32.6%)	5 (31.8%)	0.395
Female (*n* = 127)	45 (27.9%)	38 (33.8%)	38 (27.9%)	6 (33.8%)	
Marital status					
Single (*n* = 175)	55 (31.4%)	56 (32.0%)	53 (30.3%)	11 (6.3%)	0.844
Married (*n* = 16)	6 (37.5%)	6 (37.5%)	4 (25.0%)	0 (0.0%)	
Family income (ILS)					
<3,000 (*n* = 43)	9 (20.9%)	13 (30.2%)	17 (39.5%)	4 (9.3%)	0.186
3,000–5,000 (*n* = 93)	33 (35.5%)	32 (34.4%)	26 (28.0%)	2 (2.2%)	
>5,000 (*n* = 55)	19 (34.5%)	17 (30.9%)	14 (25.5%)	5 (9.1%)	
Residency					
West Bank (*n* = 119)	36 (30.3%)	39 (32.8%)	37 (31.1%)	7 (5.9%)	0.940
Other Palestinian areas (*n* = 72)	25 (34.7%)	23 (31.9%)	20 (27.8%)	4 (5.6%)	
Proficiency in English					
Novice (*n* = 20)	5 (25.0%)	7 (35.0%)	7 (35.0%)	1 (5.0%)	0.953
Intermediate (*n* = 149)	50 (33.6%)	48 (32.2%)	43 (28.9%)	8 (5.4%)	
Experienced (*n* = 22)	6 (27.3%)	7 (31.8%)	7 (31.8%)	2 (9.1%)	
Preferred pediatric clinical training modality					
Hospital (*n* = 180)	58 (32.2%)	58 (32.2%)	54 (30.0%)	10 (5.6%)	0.837
Simulation (*n* = 11)	3 (27.3%)	4 (36.4%)	3 (27.3%)	1 (9.1%)	

Values are presented as *n* (% within variable category); GAD-7, Generalized Anxiety Disorder 7-item scale. All comparisons were conducted using the Fisher–Freeman–Halton exact test, percentages are presented within each variable category.

A strong positive correlation was observed between GPA and overall Didactic pediatric grades [*ρ* = 0.678, *p* < 0.001; 95% CI (0.591, 0.750)], as well as between GPA and clinical pediatric grades [*ρ* = 0.402, *p* < 0.001; 95% CI (0.272, 0.518)]. Additionally, final exam grades in Didactic pediatric coursework correlated strongly with overall Didactic grades [*ρ* = 0.862, *p* < 0.001; 95% CI (0.819, 0.895)] and moderately with clinical pediatric grades [*ρ* = 0.246, *p* < 0.001; 95% CI (0.103, 0.378)]. The overall Didactic grade also showed a moderate relationship with clinical pediatric grades [*ρ* = 0.359, *p* < 0.001; 95% CI (0.225, 0.480)]. However, the number of simulation days exhibited no significant correlations with final exam grades (*ρ* = 0.019, *p* = 0.793), overall Didactic grades (*ρ* = 0.098, *p* = 0.177), or clinical grades (*ρ* = 0.082, *p* = 0.262). Results are presented in Table 4.

**Table 4:** Correlations between simulation exposure, GPA, and academic outcomes (*N* = 191).

Variable pair	Spearman’s *ρ*	*p*-value	95% CI lower	95% CI upper
Number of days of simulation performed for pediatric clinical—final exam grade in didactic pediatric course	0.019	0.793	−0.127	0.165
Number of days of simulation performed for pediatric clinical—overall grade in didactic pediatric course	0.098	0.177	−0.049	0.241
Number of days of simulation performed for pediatric clinical—overall grade in clinical pediatric course	0.082	0.262	−0.065	0.225
GPA—number of days of simulation performed for pediatric clinical	0.111	0.125	−0.035	0.253
GPA—overall grade in didactic pediatric course	0.678	<0.001^*^	0.591	0.750
GPA—overall grade in clinical pediatric course	0.402	<0.001^*^	0.272	0.518
Final exam grade in didactic pediatric course—overall grade in didactic pediatric course	0.862	<0.001^*^	0.819	0.895
Final exam grade in didactic pediatric course—overall grade in clinical pediatric course	0.246	<0.001^*^	0.103	0.378
Overall grade in didactic pediatric course—overall grade in clinical pediatric course	0.359	<0.001^*^	0.225	0.480

*p* < 0.001, CI, confidence interval.

**Figure 3:**
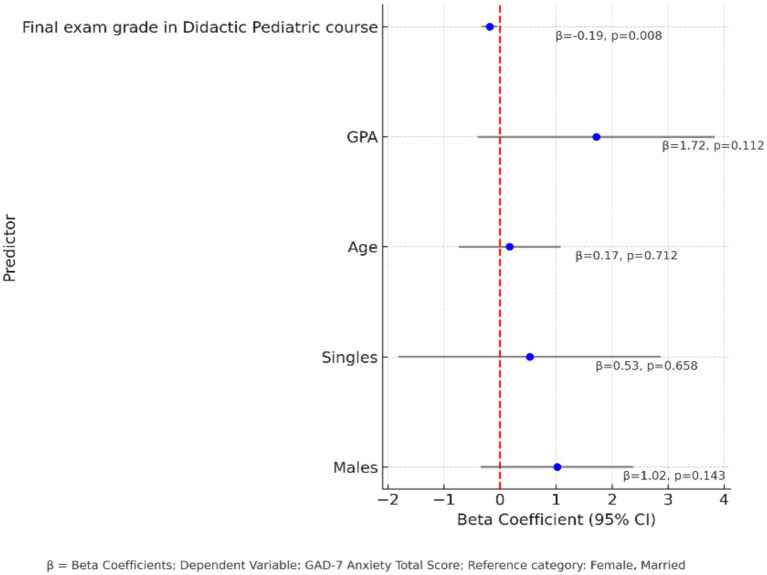
Forest plot displaying beta coefficients (95% confidence intervals) from the generalized linear model examining predictors of GAD-7 anxiety total score. The final examination grade in the didactic pediatric course was significantly associated with lower anxiety scores (*β* = −0.19, *p* = 0.008). Other predictors, including GPA, age, gender, and marital status, were not statistically significant (*p* > 0.05). Reference categories: female and married.

Figure 3 presents the results of the generalized linear model assessing the impact of demographic and academic variables on GAD-7 anxiety scores. Higher final exam grades in Didactic pediatric course were significantly associated with lower GAD-7 scores. For every one-point increase in the pediatric exam grade, anxiety scores decreased by 0.19 points. The other predictor variables: gender, marital status, age and GPA were not significantly related with anxiety scores (*p* > 0.05) respectively.

**Figure 4:**
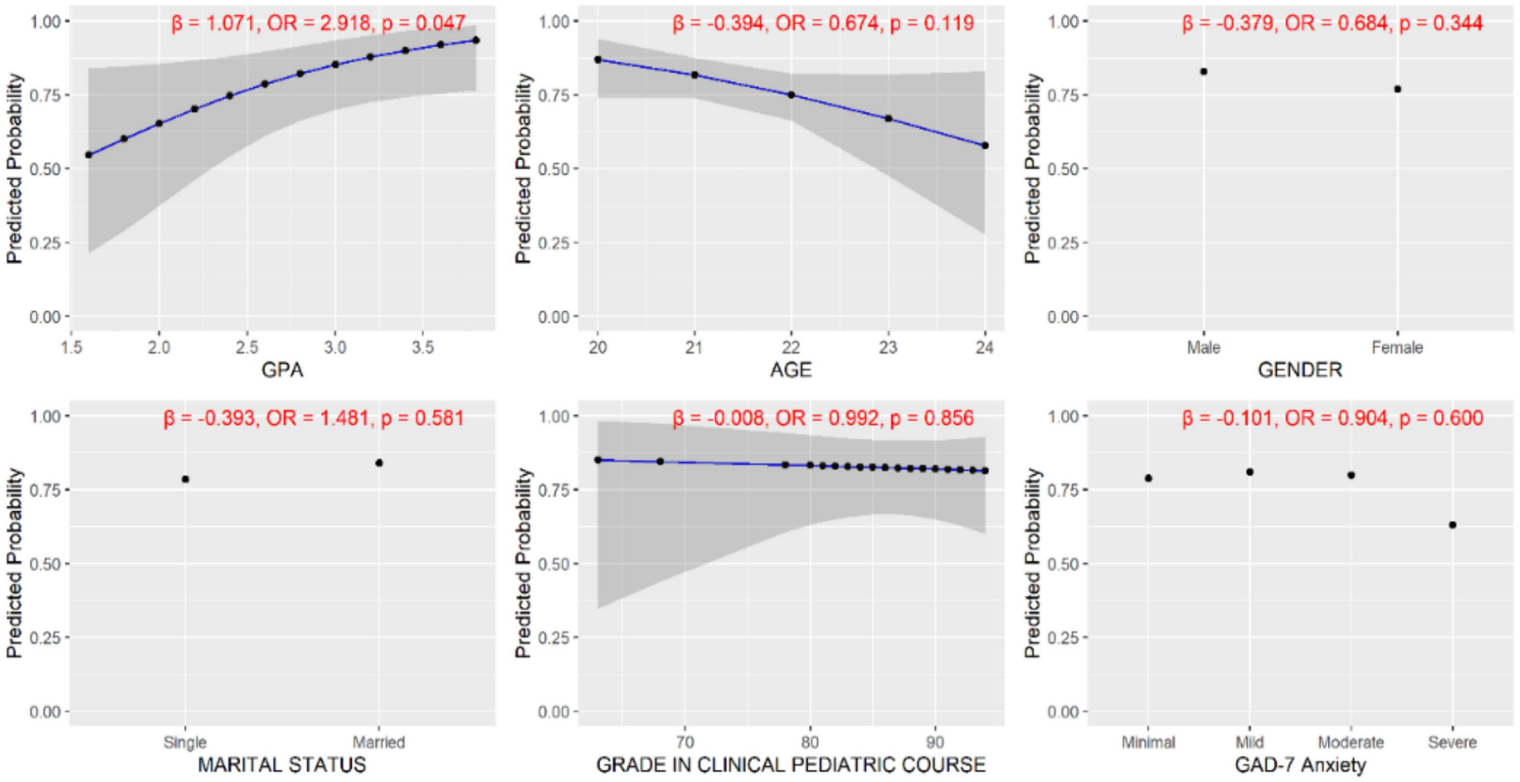
Predicted probabilities from the binary logistic regression model examining predictors of allocation to the simulation-integrated training group (reference category: hospital-only group). Each panel displays the effect of a single predictor on the probability of belonging to the simulation group, with 95% confidence intervals. Higher GPA was significantly associated with increased odds of being in the simulation group (*β* = 1.071, OR = 2.918, *p* = 0.047). Age, gender, marital status, clinical pediatric grade, and GAD-7 anxiety scores were not statistically significant predictors (*p* > 0.05).

Results presented in Figure 4 are from a binary logistic regression model examining predictors of group allocation. Students with higher GPA were 2.918 times more likely to be assigned to the simulation-integrated group compared to the traditional hospital-based group (odds ratio = 2.918, *p* = 0.047, 95% CI: 1.012–8.413). This finding suggests that academic performance may have influenced group allocation despite the absence of predefined allocation criteria, indicating a potential for selection bias. Other predictors, including age, gender, marital status, course grades, and anxiety scores, were not statistically significant.

## 4 Discussion

The present study explored the influence of simulation-integrated clinical learning on nursing students’ academic performance, satisfaction, self-confidence, and anxiety, and compared these outcomes with those of peers who completed traditional hospital-based placements. By examining academic results, psychological consequences, and learners’ perceptions, the study sought to evaluate the effectiveness and acceptability of simulation-integrated clinical learning as a potential complement to conventional clinical practicum experiences.

The findings showed that students exposed to simulation-integrated training did not differ significantly in academic performance from those trained exclusively in hospital settings. This result is consistent with previous research showing that simulation, when delivered with adequate fidelity, appropriate facilitation, and structured debriefing, may help reinforce knowledge retention and clinical reasoning skills, with small effect sizes observed in the present study (34). Comparable academic outcomes have also been documented in maternal-newborn and patient-centered care settings where simulation was incorporated into clinical curricula (35, 36). These findings add to the existing evidence suggesting that simulation may function as a pedagogically useful complement to traditional placements within similar educational contexts; however, given the non-randomized design and potential selection bias, these findings should be interpreted as associative rather than causal (37, 38).

With respect to psychological outcomes, anxiety levels did not differ significantly between students in simulation and traditional training groups. This aligns with earlier studies suggesting that simulation-based learning does not consistently reduce anxiety, particularly when essential emotional support elements such as preparatory orientation or comprehensive debriefing are insufficient (39). Similar conclusions have been drawn from related investigations showing only modest or non-significant reductions in anxiety following simulation interventions among healthcare professionals (40). Furthermore, this study found no significant associations between anxiety and demographic variables such as gender, marital status, income, or English proficiency, a finding also supported by previous literature (41). The absence of demographic influence suggests that simulation creates an emotionally equitable learning space, offering consistent opportunities for learners from diverse backgrounds (42, 43).

Beyond group comparisons, a negative relationship was identified between academic achievement and anxiety, whereby students with higher examination scores reported lower psychological distress. This observation aligns with the cognitive buffer theory, which posits that stronger academic preparation enhances an individual’s ability to cope with evaluative stress (44). Comparable trends have been documented in simulation-based research, where increased knowledge was associated with lower simulation-induced anxiety (45). Structured learning strategies such as pre-briefing, content scaffolding, and clear learning objectives may mitigate psychological strain during simulation experiences (38, 46).

These mechanisms became particularly relevant during the COVID-19 pandemic, when restricted access to clinical sites accelerated the global reliance on simulation-based learning (47). Together, these findings suggest that while simulation exposure alone may not directly reduce anxiety, academic preparedness and supportive instructional design play a critical role in moderating students’ psychological responses.

The strong positive correlation between satisfaction and self-confidence represents another key finding of this study. Students who reported greater satisfaction with simulation activities also demonstrated higher self-confidence in their clinical abilities. This result corresponds with previous findings indicating that learner satisfaction serves as a significant driver of self-efficacy, motivation, and skill retention in simulation-based education (48). Elements such as facilitator engagement, feedback quality, and emotional realism are consistently cited as essential to promoting learner satisfaction (49). Among these, debriefing has been highlighted as a compelling component, enhancing reflective reasoning and emotional integration following simulation experiences (50).

However, it is noteworthy that only approximately half of the participants expressed clear agreement regarding the effectiveness of the simulation-based instructional approach, a level of agreement that appears lower than that commonly reported in the literature. Several contextual factors may explain this discrepancy. First, simulation in the present study was integrated with hospital-based practice rather than implemented as a stand-alone immersive intervention, which may have influenced students’ comparative perceptions. Second, a strong preference for traditional hospital placements was observed among students, suggesting that direct patient interaction remains highly valued. Third, the wide variation in simulation exposure (2–21 days) may have resulted in heterogeneous learning experiences. These factors may help explain the moderate levels of agreement despite overall comparable academic and psychological outcomes. Collectively, these findings underscore the importance of designing simulations that prioritize learner engagement, emotional safety, and contextual realism rather than focusing solely on technical fidelity.

Interestingly, simulation exposure, whether expressed as days or total hours, showed no significant relationship with self-confidence, satisfaction, or anxiety. This result mirrors findings from previous studies suggesting that the frequency of simulation sessions alone does not determine learning outcomes (51). Instead, the quality of scenarios, instructional design, and facilitator effectiveness may represent more influential determinants of learning success (52). The broad range of simulation exposure in the present sample (2–21 days) may have limited the ability to detect an apparent dose–response effect. Moreover, when simulation sessions are inconsistently aligned with curricular goals or lack standardized delivery, their educational impact may be substantially diminished (53). These findings reinforce the importance of standardized simulation frameworks and consistent pedagogical delivery to ensure stable learning outcomes across student cohorts (54).

The absence of significant differences between the simulation-integrated and traditional groups does not contradict the emphasis on instructional quality. Importantly, adjusted analyses accounting for baseline differences in age, hospital attendance days, and GPA similarly demonstrated no statistically significant independent effect of training modality on academic or anxiety outcomes, reinforcing the associational interpretation of these findings. An important methodological consideration relates to the allocation process and the potential for selection bias. Although clinical placement was not based on predefined academic criteria, logistic regression analysis demonstrated that students with higher GPA were significantly more likely to be assigned to the simulation-integrated group. This finding suggests the presence of unintended or implicit allocation mechanisms, possibly related to administrative or scheduling practices. As a result, baseline differences in academic performance may have influenced group composition. While statistical adjustments were performed, residual confounding cannot be excluded, and the observed similarities in academic and psychological outcomes should therefore be interpreted cautiously.

Within this context, both groups completed equivalent total clinical hours within structured curricula and were supervised by qualified faculty, suggesting that instructional quality across settings was likely comparable. Moreover, the lack of significant correlations between simulation exposure and academic or psychological outcomes indicates that exposure quantity alone did not determine learning outcomes. Together, these findings suggest that well-designed simulation may achieve outcomes comparable to traditional hospital placements within similar institutional contexts; however, these findings should be interpreted as associative rather than evidence of equivalence or causality given the non-randomized design.

Despite similar academic and psychological outcomes, a clear preference emerged among students for traditional hospital-based clinical placements. This preference likely reflects the perception that real-world clinical environments offer higher authenticity, emotional unpredictability, and complexity. Consistent with prior research, students often regard bedside interactions and direct patient care as vital for developing interpersonal skills, empathy, and professional identity (55). Even high-fidelity simulations, while realistic, may not fully replicate the ethical, emotional, and social ambiguity of actual clinical practice (49). This contrast between objectively comparable outcomes and subjective learner preferences suggests the need for more emotionally immersive simulations that incorporate interprofessional collaboration, moral complexity, and patient-centered narratives to bridge this experiential gap (56).

Finally, a statistically significant association between GPA and simulation placement was observed, suggesting that clinical assignment processes may have varied across performance levels, even in the absence of predefined academic allocation criteria. While performance-based allocation may seem practical, it can inadvertently limit equitable access to simulation opportunities. Recent educational literature emphasizes that fairness and inclusivity should guide clinical placement policies, ensuring that all students regardless of academic ranking benefit from high-quality learning experiences (50, 57). Transparent placement criteria and equitable access to simulation are therefore essential to maintain justice, inclusivity, and pedagogical integrity in nursing education.

This study has several limitations that should be acknowledged. The non-random group allocation introduces potential selection bias. Although assignment was determined by logistical and administrative constraints rather than predefined academic criteria, logistic regression analysis demonstrated that students with higher GPA were significantly more likely to be allocated to the simulation-integrated group. This finding suggests the presence of unintended or implicit selection mechanisms, which may have influenced baseline group comparability and outcome interpretation. The cross-sectional design and non-randomized sampling restrict the ability to infer causality between simulation-integrated exposure and outcomes. In addition, the relatively small size of the traditional hospital-based group may have reduced statistical power to detect small to moderate effects. The presence of borderline *p*-values for primary outcomes and confidence intervals for most primary outcomes crossing zero suggests that some non-significant findings may be attributable to type II error rather than true absence of differences. Accordingly, these non-significant findings should be interpreted with caution and should not be considered evidence of equivalence or absence of true differences between training modalities.

Variation in the number of simulation days (ranging from 2 to 21) may have influenced the comparability of experiences across participants. Including students with limited simulation exposure may have attenuated potential differences between groups, thereby contributing to the absence of detectable dose–response effects. In addition, reliance on self-reported instruments for satisfaction, confidence, and anxiety introduces potential response bias. Prior informal healthcare experience and previous exposure to simulation-based learning were not specifically measured and may have influenced learner perceptions and outcomes. Although all participants followed a standardized curriculum within the same institution, unmeasured variability in baseline exposure cannot be entirely excluded. The single-institution setting further limits the generalizability of findings to broader nursing populations. Future studies should employ randomized controlled or longitudinal designs to explore causal relationships and long-term impacts of simulation on clinical competence and professional practice. Implementing standardized simulation frameworks, faculty training, and consistent debriefing protocols could enhance educational quality and comparability. Multi-institutional research across diverse cultural and resource settings is also recommended to validate the transferability of these findings.

The findings of this study carry important implications for nursing education, particularly in environments where access to clinical placements is restricted. The absence of statistically significant differences in academic and psychological outcomes suggests that simulation-integrated training may serve as a potentially viable complementary strategy and, under structured conditions, may help mitigate gaps in clinical placement availability without evidence of educational disadvantage within this context.

Faculty and curriculum planners should focus on improving simulation design rather than merely increasing its frequency. Enhancing scenario realism, ensuring structured debriefing, and fostering emotional engagement can help maximize learner satisfaction and self-confidence. The absence of demographic disparities in anxiety outcomes suggests that simulation-based learning did not disadvantage specific student subgroups within this sample. While broader conclusions regarding inclusivity require further investigation, these findings highlight the importance of maintaining transparent placement policies to ensure fair access to simulation opportunities for all students. Addressing students’ preferences for real-world experiences through emotionally rich, socially interactive, and contextually realistic simulations, may further enhance preparedness of nursing graduates for contemporary healthcare practice.

## 5 Conclusion

Immersive simulation-integrated clinical education may serve as a pedagogically useful complement to traditional hospital placements, with no statistically significant differences detected in most measured academic, satisfaction, self-confidence, or anxiety outcomes among undergraduate nursing students in this cohort. The absence of significant differences in anxiety across demographic subgroups suggests that simulation-based learning environments may provide a supportive and inclusive educational setting. Although the number of simulation days did not directly influence outcomes, the strong positive relationship between satisfaction and self-confidence highlights the central role of thoughtful simulation design and facilitation quality. Despite these findings, students continued to express a preference for traditional clinical experiences, suggesting that future simulation programs should strive to strengthen emotional authenticity, social interaction, and contextual realism. Overall, the results support the thoughtful integration of simulation within nursing curricula, particularly in settings where access to clinical placements is limited; however, these findings should not be interpreted as evidence of equivalence or causality given the non-randomized design and baseline group differences.

## Data Availability

The raw data supporting the conclusions of this article will be made available by the authors, without undue reservation.
