# Supplementary material for: Immersive clinical learning using simulation and its impact on academic performance, satisfaction, self-confidence, and anxiety among pediatric nursing students
Source: Front Med (Lausanne). 2026 May 7;13:1742360. doi: 10.3389/fmed.2026.1742360 (PMC13189756; doi:10.3389/fmed.2026.1742360)
Supplement: Supplementary file 1 [file Table_1.docx]

**Supplementary Appendix A**

**Table A1. Summary of Study Instruments and Variables**

| Domain | Variable / Instrument | Description / Item Wording | Response Format / Categories | Notes |
| --- | --- | --- | --- | --- |
| Preference | Clinical training preference | “If given a choice, which clinical training modality would you prefer for pediatric practice?” | 1. Hospital-based clinical training 2. Simulation-based clinical training | Single-response categorical variable |
| Simulation Exposure | Simulation days | Number of simulation days completed | Range: 2–21 days | One day = 5-hour structured session including pre-briefing, scenario implementation, and debriefing |
|  | Simulation hours | Total hours in simulation training | Continuous variable | Derived from institutional records |
|  | Simulation proportion | Simulation hours / total clinical hours (168) | Continuous (%) | Reflects relative exposure |
| Academic Performance | GPA | Cumulative Grade Point Average | Continuous | Self-reported (verified against institutional records where applicable) |
|  | Final exam (Didactic) | Pediatric course final exam score | Percentage (%) | Theoretical knowledge |
|  | Didactic total grade | Overall pediatric didactic course score | Continuous (%) | Composite score |
|  | Clinical grade | Pediatric clinical course performance | Continuous (%) | Based on standardized rubric |
| Clinical Evaluation | Performance assessment | Faculty evaluation of clinical skills | Standardized faculty evaluation rubric | Covers clinical reasoning, technical skill performance, communication, and professional behavior; applied consistently across both training groups |
| Satisfaction & Confidence | NLN Satisfaction & Self-Confidence Scale | 13-item validated instrument | 5-point Likert (1–5) | α = 0.94 (satisfaction), 0.87 (confidence); simulation group only |
| Anxiety | GAD-7 | 7-item anxiety scale (past 2 weeks) | Score: 0–21; Categories: Minimal (0–4), Mild (5–9), Moderate (10–14), Severe (15–21) | Used as continuous + categorical |
| Demographics | Gender | Self-reported | Male / Female | — |
|  | Marital status | Self-reported | Single / Married | — |
|  | Income | Monthly household income | <3000 / 3000–5000 / >5000 ILS | — |
|  | Residency | Place of residence | West Bank / Other Palestinian areas | Categories reflect geopolitical classification used in institutional records |
|  | English proficiency | Self-assessed proficiency | Novice / Intermediate / Experienced | Relevant to instruction language |
| Clinical Training Type | Training modality | Type of clinical exposure | Simulation-integrated / Hospital-only | Non-random allocation |
